# Expression Analysis of the orphan receptors GPR161, GPR132, GPR20, and GPR139 in patients with cervicitis and low-grade, and high-grade squamous intraepithelial lesions

**DOI:** 10.1590/1678-4685-GMB-2025-0191

**Published:** 2026-03-30

**Authors:** Jessica E. Rodríguez, Javier González-Ramírez, Alina Barquet-, Gladys E. Ramírez-Rosales, Lilly G. Cruz-Izaguirre, Brenda J. Reyes-Solorio, Rosa P. Cruz-Nieves, Edgar Torres-Maravilla, Armando Ruiz-Hernández

**Affiliations:** 1 Universidad Nacional Autónoma de Mexico, Facultad de Estudios Superiores Zaragoza, Bioquímica clínica, Ciudad de México, México.; 2 Universidad Autónoma de Baja California, Centro de Ciencias de la Salud, Laboratorio de Biología Molecular, Mexicali, Baja California, Mexico.; 3 Instituto Politécnico Nacional, Escuela Superior de Medicina, Sección de Estudios de Posgrado e Investigación, Ciudad de México, Mexico.; 4 Universidad Autónoma de Baja California, Facultad de Medicina, Mexicali, Baja California, Mexico.

**Keywords:** Orphan receptors, cervicitis, intraepithelial lesions.

## Abstract

Cervical cancer is one of the most common types of carcinomas causing morbidity and mortality in women worldwide, primarily caused by high-risk human papillomavirus (hrHPV). However, some cervical tumors, particularly adenocarcinomas, may develop independently of HPV. Chronic cervicitis, often linked to bacterial infections, increases the risk of developing squamous low and high-grade intraepithelial lesions (LSIL and HSIL, respectively) and cervical cancer. Cancer-associated inflammation activates key signaling pathways (NF-κB, STAT, and FOXO), regulating orphan G protein-coupled receptors (oGPCRs) implicated in cancer. Notably, GPR161, GPR132, GPR20, and GPR139 play roles in various tumors. This study analyzed cervical tissue samples from 45 women undergoing colposcopy at UNEME Oncology in Mexicali, Baja California. Patient data, including reproductive history and lifestyle factors, were collected. The histopathological analysis identified 31.1% with cervicitis, 40% with LSIL, and 28.8% with HSIL. mRNA expression of orphan GPRs varied among groups, with GPR132 and GPR20 significantly elevated in LSIL samples (p < 0.05), while GPR139 expression was reduced in LSIL (p < 0.05). No significant difference was found for GPR161. The differential expression of oGPCRs in cervical tissue suggests their involvement in the progression of LSIL and HSIL, offering insights into novel therapeutic targets.

## Introduction

Cervical cancer remains a significant health concern, ranking among the most prevalent cancers affecting women worldwide. In 2020 alone, there were an estimated 604,127 new cases and 341,831 deaths globally (Singh et al., 2023). While persistent infection with one of approximately 15 high-risk human papillomavirus (hrHPV) types is a well-established cause of cervical cancer (Bosch et al., 2002), increasing evidence suggests that some cervical tumors, particularly adenocarcinomas, may not be linked to HPV infection (Park, 2020). In this sense, chronic inflammation of the cervix (cervicitis), can lead to several adverse outcomes, including pelvic inflammatory disease, endometriosis, infertility, and preterm birth. While cervicitis is often attributed to bacterial infections, the presence of hrHPV sequences has been detected in some cases. Women experiencing cervicitis concurrently with HPV infection appear to have an elevated risk of developing low and high-grade squamous intraepithelial lesions (LSIL and HSIL, respectively) and, ultimately, cervical cancer (Koutsky et al., 1992; Fernandes et al., 2015). Cancer-related chronic inflammation promotes the activation of intracellular signaling pathways, which in turn induce numerous transcription factors, including NF-κB, STAT, and FOXO (Zhao et al., 2021; Kim et al., 2022). These transcription factors modulate the expression of a wide range of genes, including those encoding orphan G protein-coupled receptors (oGPCRs). GPCRs are cell-surface receptors that transmit extracellular signals into intracellular pathways by activating heterotrimeric G proteins. While the endogenous ligands for many orphan GPCRs remain unidentified, their importance in various diseases, including cancer, has been clearly demonstrated (Tang et al., 2012). For instance, GPR161, a negative regulator of Sonic hedgehog (Shh) signaling during neural tube development, has been shown to play a role in medulloblastoma. In mouse neural stem cells, suppressing GPR161 increased downstream Shh pathway activity, leading to increased granule cell generation and proliferation, ultimately contributing to higher tumor incidence and medulloblastoma pathogenesis (Shimada et al., 2018). GPR132 functions as a key macrophage sensor, detecting elevated lactate levels within the acidic tumor microenvironment. This sensing plays a crucial role in mediating the reciprocal interactions between cancer cells and macrophages during breast cancer metastasis (Chen et al., 2017). Also, GPR20 has been identified as a non-tyrosine kinase target in gastrointestinal stromal tumor (GIST) (Iida et al., 2021); meanwhile, the GPR139 gene has been identified in glioblastoma multiforme samples (Ren et al., 2021).

Therefore, this study investigated the expression of the orphan GPR161, GPR132, GPR20, and GPR139 in samples of cervicitis, LSIL, and HSIL to elucidate their potential roles in the pathogenesis of cervical cancer. It is important to mention that a significant portion of the tissue samples used to carry out this study were previously used to analyze the expression of nuclear orphan receptors of the NR4A family and due to the results obtained by the working group, a search was carried out for other probable biological markers in the development of cervical cancer, obtaining new data and not published interesting results that will be analyzed and discussed in this article (Cruz et al., 2024).

## Subjects and Methods

### Recruitment of patients and sample collection

This study was approved by the Ethics and Research Committee of the Maternity Hospital of Mexicali (registration number CDEI-0008-21). All participants provided written informed consent before enrollment. Between January 2022 and January 2023, 45 patients were recruited from the Medical Oncology Specialties Unit (UNEME) in Mexicali, Baja California, Mexico. Fresh cervical tissue samples were collected during colposcopy. Each sample was divided, with one portion used for histopathological analysis and the other stored at -80 °C for subsequent gene expression analysis (Cruz et al., 2024).

### Total RNA extraction and cDNA synthesis

Total RNA was extracted from the cervical tissue samples using TRIzol® Reagent (Thermo Fisher Scientific, USA) following the manufacturer’s protocol. The concentration and purity of the RNA were determined by measurement of the optical densities at 260 nm and 280 nm using a NanoDrop 1000 spectrophotometer (Thermo Fisher Scientific, USA). An A260/A280 ratio of 1.8 or higher were considered acceptable for these studies. These RNA samples were then diluted to a working concentration of 1 µg/mL in nuclease-free water supplemented with RNase inhibitor. For the reverse transcription synthesis of cDNA, the iScript cDNA synthesis kit (Bio-Rad, USA) was used according to the manufacturer’s instructions (Cruz et al., 2024).

### Determination of mRNA expression of orphan receptors

The primers used for PCR targeted GPR161*, GPR132, GPR20, GPR139* and *GADPH* (Table 1). Relative mRNA expression levels were determined using the comparative ΔΔCt method.

**Table 1 -:** Primer sequences used in this study**.**

Receptor	Primers	Sequences	Tm(°C)
GPR20	Fw	GTGTCTTTGCGCTGACTGTC	56.6
	Rs	ATGATGCGGCCGGTAAACA	57.5
GPR132	Fw	CGGAAGACAAGGAGACCTGC	50.1
	Rs	CGGTCAACCTGGCGTAGTAG	50.3
GPR139	Fw	ATTGCCAACATGCTAGCCCT	57.3
	Rs	GGAACCGCTTGCTGATGAAG	56.6
GPR161	Fw	CCTTGGGAGCATGTCACTGT	57.4
	Rs	CTTGTCCTGGTGGCTGCATA	57.4
GAPDH	Fw	CATCCTGGGCTACACTGAGC	57.7
	Rs	GTCAAAGGTGGAGGAGTGGG	57.7

The RNA extracted from cervix was reverse transcribed and used for real-time PCR analysis with the QuantStudioTM 1 system (Thermo Fisher Scientific, USA). The reaction mixture for PCR consisted of 5 µL of PanGreen Universal Master Mix (Bio-Helix Ltd, Taiwan), 0.3 µL of forward primer (Integrated DNA Technologies, USA), 0.3 µL of reverse primer (Integrated DNA Technologies, USA), 3.4 µL of PCR-grade water, and 500 ng of cDNA template (Cruz et al., 2024).

### Statistical analysis

Results are presented as mean ± standard error. Statistical analysis was performed using the Kruskal-Wallis test. A p-value of less than 0.05 was considered statistically significant. All analyses were conducted using GraphPad Prism (version 8.0.1).

## Results

### Patients

In this study, 45 tissue samples from cervices were collected from women who underwent colposcopy and biopsy. The samples were collected from patients aged between 15 and 60 years, with an average age of 35 years. The average age of menarche onset was 12 years (Cruz et al., 2024). Regarding the beginning of sexual activity, it had occurred at 17 years of age on average, and 22% of the women had reported more than three sexual partners (Cruz *et al*., 2024). The use of contraceptive methods, salpingoplasty and condoms were the most used by patients (Cruz *et al*., 2024). In addition, other important factors that could have affected the health of the women in this study were the following: Five patients were smokers, 14 were alcoholics, and one patient had reported cocaine use. On the other hand, the body mass index indicated that 75.5% of them had body mass indexes >30, which was compatible with overweight and obesity (Table 2) (Cruz *et al*., 2024).

**Table 2 -:** Demographic and clinical characteristics.

Variable	n = 45	
*Age (years)*		
Mean	35	
Minimum	15	
Maximum	60	
*Menarche*		
Mean	12	
Minimum	9	
Maximum	17	
*Onset of active sexual life*		
Mean	17	
Minimum	15	
Maximum	20	
*Sexual partners*		
1 to 3	35	
> 3	10	
*Body mass index*	No.	%
Low weight	0	0
Normal	11	24.4
Overweight	10	22.2
Obesity	24	53.3
*Contraceptive methods*		
Hormonal	15	33.3
Condom	5	11.1
Salpingoplasty	25	55.5
*Pap test*		
ASCUS	13	28.8
CIN I	21	46.6
CIN II	1	2.2
CIN III	7	15.5
Other	3	6.6
*Colposcopy*		
Cervicitis	14	31.1
LSIL	18	40
HSIL	13	28.8

ASCUS = atypical squamous cells of undetermined significance; CIN = cervical intraepithelial neoplasia; LSIL = low-grade squamous intraepithelial lesions; HSIL = high-grade squamous intraepithelial lesions.

The results obtained from the cervical cytology were benign (atrophy or inflammation) in 6.6% of cases, ASCUS (atypical cells of uncertain significance) in 28.8%, cervical intraepithelial neoplasia 1(CIN I; low-grade dysplasia that affects up to one-third of the cervical lining) in 46.6%, cervical intraepithelial neoplasia 2(CIN II; moderate to marked dysplasia that affects up to two-thirds of the cervical lining) in 2.2%, and cervical intraepithelial neoplasia 3 (CIN III; severe dysplasia to carcinoma *in situ* that affects the full thickness of the cervical lining) in 15.5% of the patients. The tissue analysis of HSIL confirmed high-grade lesions, as indicated by the cytological findings, in five cases (CIN II or CIN III). (Table 3). Cervical samples for one year, 31.1% exhibited acute and chronic non-specific endocervicitis and exocervicitis, 40% exhibited LSIL, and 28.8% HSIL (Cruz et al., 2024).

**Table 3 -:** Cervical biopsy results related to cytology results.

HPR	Results of cervical cytology				
	Benign findings	ASCUS	CIN I	CIN II	CIN III
Cervicitis	12.5% (n = 2)	37.5% (n = 6)	50% (n = 6)	0	0
LSIL	10.5% (n = 1)	26.3% (n = 5)	52.6% (n=9)	0	10.5% (n = 3)
HSIL	0	10% (n = 2)	70% (n = 6)	10% (n = 1)	10% (n = 4)

HPR = histopathological result; ASCUS = atypical squamous cells of undetermined significance; CIN = cervical intraepithelial neoplasia; LSIL = low-grade squamous intraepithelial lesions; HSIL = high-grade squamous intraepithelial lesions ^[13]^.

### mRNA expression of GPR161, GPR132, GPR20, and GPR139 orphan receptors

**Figure 1 -:**
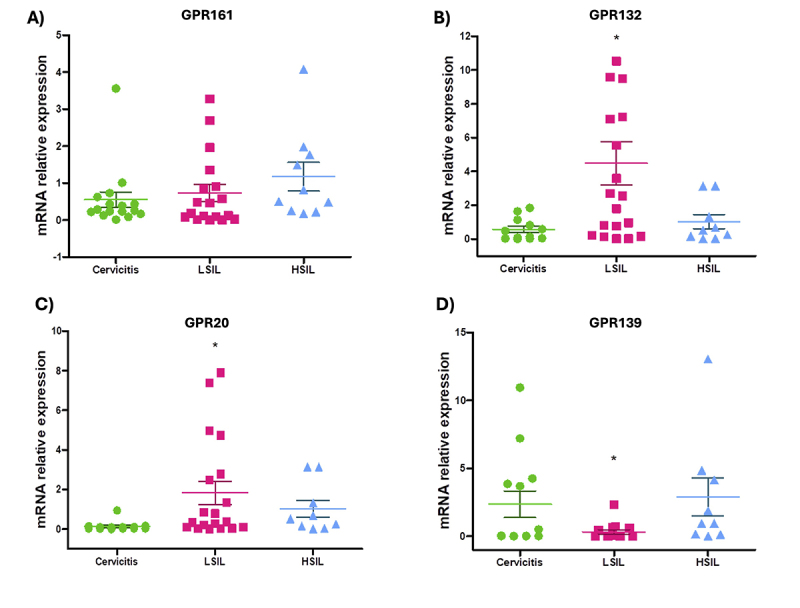
mRNA expression of the oGPRs. (A) GPR161, (B) GPR132, (C) GPR20, and (D) GPR139 receptors in cervical biopsy samples: Cervicitis (n=14), LSIL (n=18) and HSIL (n=13). Data were represented as mean ± SEM. *p < 0.05.

Orphan receptors were expressed in cervical samples; however, their expression changed due to the development of cervicitis or LSIL/HSIL lesions. While mRNA expression of GPR161 showed no statistically significant difference among the three groups (p > 0.05), mRNA of GPR132 was increased in LSIL samples compared to both cervicitis and HSIL patients (p < 0.05). Similarly, the mRNA of GPR20 was higher in the LSIL group compared to the cervicitis and HSIL groups (p < 0.05). Conversely, GPR139 had diminished expression in the LSIL group, a difference that was statistically significant (p < 0.05) compared with the other two sample groups (Figure 1).

## Discussion

The role of chronic inflammation in epithelial carcinogenesis is well-established. Studies have demonstrated a positive correlation between cervicitis and squamous intraepithelial lesions (SILs) (Koutsky et al., 1992; Castle et al., 2001). SILs are categorized into low-grade (LSIL) and high-grade lesions (HSIL), based on their potential for progression to carcinoma. While LSIL is generally considered a low-risk precursor to cervical cancer, it is established that both low- and high-risk HPV can infect a broad range of cervical cell types, including columnar, reserve, mature, intermediate, and immature squamous cells (Pinto et al., 2010), producing the progression from LSIL to HSIL, and later cervical cancer due to the interactions of several viral oncoproteins that dysregulate cell signaling pathways involved in proliferation, DNA damage, or repair mechanisms (Manzo-Merino et al., 2014). Among the cellular changes observed in carcinogenesis, the modification in the expression and activity of the GPCRs, including orphan receptors, has been described. Although oGPCRs lack identified endogenous ligands (Jobe and Vijayan 2024), they exhibit therapeutic potential in the treatment of metabolic disorders and autoimmune diseases (Jobe and Vijayan 2024). But, specifically in cancer, they have been implicated in cellular growth and tumor progression, making them promising targets for novel therapeutic interventions. Therefore, the present study aimed to examine the mRNA expression of selected oGPCRs that have been previously implicated in other types of cancer, but whose role in cervicitis and SIL, precursors to cervical cancer, remains unexplored. 

Our results showed that mRNA of oGPCRs (GPR161, GPR132, GPR20, and GPR139) are expressed in cervical tissue, but their expression differed across cervicitis, LSIL, and HSIL samples. Notably, GPR132 mRNA was significantly increased in LSIL samples compared to both cervicitis and HSIL samples. This overexpression of GPR132 in LSIL samples may represent an intrinsic compensatory mechanism to mitigate hyperactivation of the PI3K/AKT/mTOR pathway, given the role of GPR132 activation in promoting myeloid differentiation. Some experiments have demonstrated that the natural agonist 8-gingerol activates the GPR132-Gs-PKA pathway, leading to the inhibition of mTOR signaling, a critical suppressor of cell differentiation in acute myeloid leukemia (AML). This inhibition resulted in reduced tumor growth, increased cell differentiation, and extended mouse survival in an AML xenograft model (Yi et al., 2022). While the PI3K/AKT/mTOR pathway is crucial for physiological processes like cell survival and proliferation, its aberrant activation contributes to tumor development (Lim et al., 2015). In cervical cancer, HPV oncoproteins E6 and E7 stimulate mTOR through the PI3K/Akt signaling cascade. This pathway exhibits a high mutation rate in the PIK3CA gene in samples of cervical cancer, adenocarcinoma, and squamous cell carcinomas. The loss of the phosphatase and tensin homolog (PTEN), a key tumor suppressor and negative regulator of this pathway, frequently underlies this dysregulation (Bahrami et al., 2017). This fact is particularly relevant considering the common progression from LSIL to HSIL and finally cervical cancer, a process influenced by the interactions of several viral oncoproteins that dysregulate cell signaling. This compensatory mechanism could be crucial in early-stage disease.

Additionally, our results demonstrated that the mRNA of GPR20 was expressed in cervical tissue, with higher expression levels observed in LSIL samples compared to cervicitis and HSIL samples. GPR20 is a ligand-independent oGPCR, 358 amino acids in length, that exhibits constitutive activity and is coupled to Gi protein. Its expression is controlled by the transcription factors Forkhead box F1 (FOXF1) and ETS variant transcriptor factor 1 (ETV1) (Iida et al., 2021). GPR20 is highly expressed in intestinal tissue and is found with great abundance in gastrointestinal stromal tumors (Iida *et al*., 2021). Molecular dynamics simulations demonstrated that GPR20 exhibits high basal activity attributed to a non-canonical conformation of transmembrane helix 7 (TM7); this conformation brings the N-terminal cap into the helix center, promoting its self-interaction with Gi protein, which makes it a highly stimulated receptor. Consequently, the N-terminal cap acts as an intrinsic agonist modulating the receptor’s signal transduction and dynamic behavior (Zhang et al., 2025). Although GPR20 is a receptor that is highly expressed in GIST tumors, its role in cervical cancer is not known. However, it has already been seen that GPR20 does not have an impact on the proliferation of GIST cells, but its blockade is a potential therapeutic target for this disease (Iida *et al*., 2021). Considering that GPR20 is regulated by ETV1, its likely implication in cancer is related to angiogenesis, given that ETV1 belongs to the ETS family of transcription factors, which contributes to cancer progression, activating responses of promoters to the Ras/Raf7MEK/ERK1/2 pathways (Dittmer 2015).

Contrarily, the mRNA expression of GPR139 was reduced in LSIL samples. GPR139 activates several G protein pathways, with Gq/11 being the most important. For this receptor, it has been proposed that the aromatic amino acids L-Trp and L-Phe, as well as ACTH/α-MSH-related peptides, are endogenous agonists (Vedel et al., 2020). While the mRNA of GPR139 is predominantly expressed in the central nervous system in humans, rats, and mice (Wang et al., 2019), we detected its expression in human cervical tissue. GPR139 has been implicated in several central nervous system mechanisms, including opioid modulation by opposing μ-opioid receptor activity (Stoveken et al., 2020), and has been proposed as a therapeutic target for schizophrenia and drug addiction (Mao et al., 2023). However, its role in cancer remains poorly understood, but it is already known that the activation of GPR139 is dependent on mitogen-activated protein kinase-ERK phosphorylation (Liu *et al*., 2015), the key mechanism involved in the development of malignant tumors in cervical squamous cancer tissues (Li *et al*., 2020). Finally, despite we detected GPR161 mRNA in cervical tissue, we observed no significant differences in its expression across cervicitis, LSIL, and HSIL samples. This suggests that GPR161 expression is not modulated during the development of these cervical lesions and may not contribute to their progression.

## Conclusions

GPR132, GPR20, and GPR139 expression was modified in LSIL samples compared to cervicitis and HSIL samples. Given the involvement of the signalling pathway of these receptors in several types of cancer, we proposed that their differential regulation in LSIL may represent a mechanism to prevent the progression to HSIL and later cervical cancer. Therefore, further exploration of oGPCRs could be crucial for identifying novel therapeutic targets for cervical cancer treatment.

## Data Availability

 The data that support the findings of this study are available from the corresponding author [ARH] on reasonable request.

## References

[B1] Bahrami A, Hasanzadeh M, Hassanian SM, ShahidSales S, Ghayour-Mobarhan M, Ferns GA, Avan A (2017). The potential value of the PI3K/Akt/mTOR signaling pathway for
assessing prognosis in cervical cancer and as a target for
therapy. J Cell Biochem.

[B2] Bosch FX, Lorincz A, Muñoz N, Meijer CJLM, Shah KV (2002). The causal relation between human papillomavirus and cervical
cancer. J Clin Pathol.

[B3] Castle P, Hillier S, Rabe L, Hildesheim A, Herrero R, Bratti M, Sherman M, Burk R, Rodriguez AC, Alfaro M (2001). An association of cervical inflammation with high-grade cervical
neoplasia in women infected with oncogenic human papillomavirus
(HPV). Cancer Epidemiol Biomarkers Prev.

[B4] Chen P, Zuo H, Xiong H, Kolar MJ, Chu Q, Saghatelian A, Siegwart DJ, Wan Y (2017). Gpr132 sensing of lactate mediates tumor-macrophage interplay to
promote breast cancer metastasis. Proc Natl Acad Sci U S A.

[B5] Cruz RP, Ramírez GE, Gonzalez J, Sánchez F, Ruiz A (2024). Analysing the gene expression profiles of the orphan nuclear
receptors NR4A1, NR4A2 and NR4A3 in premalignant lesions of the cervix and
cervicitis. Eur J Obstet Gynecol Reprod Biol X.

[B6] Dittmer J (2015). The role of the transcription factor ets1 in
carcinoma. Semin Cancer Biol.

[B7] Fernandes JV, Fernandes TAADM, de Azevedo JCV, Cobucci RNO, de Carvalho MGF, Andrade VS, De Araújo JMG (2015). Link between chronic inflammation and human
papillomavirus-induced carcinogenesis (Review). Oncol Lett.

[B8] Iida K, Ahmed AHA, Nagatsuma AK, Shibutani T, Yasuda S, Kitamura M, Hattori C, Abe M, Hasegawa J, Iguchi T (2021). Identification and therapeutic targeting of GPR20, selectively
expressed in gastrointestinal stromal tumors, with DS-6157a, a
first-in-class antibody-drug conjugate. Cancer Discov.

[B9] Jobe A, Vijayan R (2024). Orphan g protein-coupled receptors: the ongoing search for a
home. Front Pharmacol.

[B10] Kim ME, Kim DH, Lee JS (2022). Transcription factors as targets of natural compounds in
age-related diseases and cancer: Potential therapeutic
applications. Int J Mol Sci.

[B11] Koutsky LA, Holmes KK, Critchlow CW, Stevens CE, Paavonen J, Beckmann AM, DeRouen TA, Galloway DA, Vernon D, Kiviat NB (1992). A cohort study of the risk of cervical intraepithelial neoplasia
grade 2 or 3 in relation to papillomavirus infection. N Engl J Med.

[B12] Li J, Rong X, Liu Y, Du J (2020). Expression of PI3K and ERK in Uygur and Han patients with
cervical squamous cancer. Int J Clin Exp Pathol.

[B13] Liu C, Bonaventure P, Lee G, Nepomuceno D, Kuei C, Wu J, Li Q, Joseph V, Sutton SW, Eckert W (2015). GPR139, an orphan receptor highly enriched in the habenula and
septum, is activated by the essential aminoacids L-Tryptophan and
L-Phenylalanine. Mol Pharmacol.

[B14] Lim HJ, Crowe P, Yang JL (2015). Current clinical regulation of PI3K/PTEN/Akt/mTOR signalling in
treatment of human cancer. J Cancer Res Clin Oncol.

[B15] Manzo-Merino J, Contreras-Paredes A, Vázquez-Ulloa E, Rocha-Zavaleta L, Fuentes-Gonzalez AM, Lizano M (2014). The Role of signaling pathways in cervical cancer and molecular
therapeutic targets. Arch Med Res.

[B16] Mao J, Cui Y, Wang H, Duan W, Liu ZJ, Hua T, Zhou N, Cheng J (2023). Design and synthesis of novel GPR139 agonists with therapeutic
effects in mouse models of social interaction and cognitive
impairment. J Med Chem.

[B17] Park KJ (2020). Cervical adenocarcinoma: integration of HPV status, pattern of
invasion, morphology and molecular markers into
classification. Histopathology.

[B18] Pinto AP, Crum CP, Hirsch MS (2010). Molecular markers of early cervical neoplasia. Diagn Histopathol (Oxf).

[B19] Ren P, Wang JY, Li L, Lin XW, Wu GH, Chen JY, Zeng ZR, Zhang HM (2021). Identification of key genes involved in the recurrence of
glioblastoma multiforme using weighted gene co-expression network analysis
and differential expression analysis. Bioengineered.

[B20] Shimada IS, Hwang SH, Somatilaka BN, Wang X, Skowron P, Kim J, Kim M, Shelton JM, Rajaram V, Xuan Z (2018). Basal suppression of the sonic hedgehog pathway by the
g-protein-coupled receptor gpr161 restricts medulloblastoma
pathogenesis. Cell Rep.

[B21] Singh D, Vignat J, Lorenzoni V, Eslahi M, Ginsburg O, Lauby-Secretan B, Arbyn M, Basu P, Bray F, Vaccarella S (2023). Global estimates of incidence and mortality of cervical cancer in
2020: A baseline analysis of the WHO global cervical cancer elimination
initiative. Lancet Glob Health.

[B22] Stoveken HM, Zucca S, Masuho I, Grill B, Martemyanov KA (2020). The orphan receptor GPR139 signals via Gq/11 to oppose opioid
effects. J Biol Chem.

[B23] Tang XL, Wang Y, Li DL, Luo J, Liu MY (2012). Orphan G protein-coupled receptors (GPCRs): Biological functions
and potential drug targets. Acta Pharmacol Sin.

[B24] Vedel L, Nøhr AC, Gloriam DE, Bräuner-Osborne H (2020). Pharmacology and function of the orphan GPR139 g protein-coupled
receptor. Basic Clin Pharmacol Toxicol.

[B25] Wang L, Lee G, Kuei C, Yao X, Harrington A, Bonaventure P, Lovenberg TW, Liu C (2019). GPR139 and dopamine D2 receptor co-express in the same cells of
the brain and may functionally interact. Front Neurosci.

[B26] Yi C, He J, Huang D, Zhao Y, Zhang C, Ye X, Huang Y, Nussinov R, Zheng J, Liu M (2022). Activation of orphan receptor GPR132 induces cell differentiation
in acute myeloid leukemia. Cell Death Dis.

[B27] Zhang MY, Ao JY, Liu N, Chen T, Lu SY (2025). Exploring the constitutive activation mechanism of the class A
orphan GPR20. Acta Pharmacol Sin.

[B28] Zhao H, Wu L, Yan G, Chen Y, Zhou M, Wu Y, Li Y (2021). Inflammation and tumor progression: signaling pathways and
targeted intervention. Signal Transduct Target Ther.

